# Primary Pulmonary Leiomyosarcoma: An Unusual Cause of Pleural Effusion

**DOI:** 10.7759/cureus.23821

**Published:** 2022-04-04

**Authors:** Syed Hamza Bin Waqar, Biplov Adhikari, Navid Salahi, Sara Ahmed, Sadafsadat Mirkarimi

**Affiliations:** 1 Internal Medicine, State University of New York Downstate Health Sciences Center, New York, USA; 2 Internal Medicine, MedStar Union Memorial Hospital, Baltimore, USA; 3 Pathology, State University of New York Downstate Health Sciences Center, New York, USA; 4 Internal Medicine, Dow University of Health Sciences, Karachi, PAK; 5 Anesthesiology, University of Minnesota, Minneapolis, USA

**Keywords:** exudative, pleural effusion, pleural mass, lung, leiomyosarcoma, lms, ppl, primary pulmonary leiomyosarcoma

## Abstract

Primary pulmonary leiomyosarcomas (PPLs) are extremely rare tumors of the lungs. They can present with non-specific symptoms or can also be asymptomatic with clues towards diagnosis being found on routine examination or radiographs. We present a case of a 54-year-old woman who presented with worsening shortness of breath and spells of dizziness. Her chest radiographs showed right-sided pleural effusion and CT revealed a large enhancing pleural mass with compression atelectasis and mediastinal shift. She underwent a thoracoscopy and right pleural biopsy. Histopathology and immunohistochemistry were most consistent with leiomyosarcoma. An extensive search for a possible primary in other sites was unrevealing, thus diagnosing the patient with PPL. She was managed with surgery and radiotherapy.

## Introduction

Primary sarcomas of the lungs are exceedingly rare tumors. They comprise less than 0.5% of all lung malignancies [1]. These malignant tumors can vary according to histopathology - the common histologic types described are angiosarcoma, rhabdomyosarcoma, hemangiopericytoma, fibrosarcoma, and leiomyosarcoma [2]. Primary pulmonary leiomyosarcomas (PPL) are the most common type of primary sarcomas of the lungs. They represent 30% of the primary sarcomas of the lungs [3]. Davidsohn, in 1903, described these elusive tumors for the first time [4]. However, their clinicopathological characteristics remain not well understood. We describe a case of PPL in a woman who initially presented with worsening shortness of breath and spells of dizziness.

## Case presentation

A 54-year-old woman with a known past medical history of diabetes mellitus presented with worsening shortness of breath and dizzy spells for the past few days. On initial presentation, the patient had tachypnea to 29 breaths per minute, tachycardia to 110 beats per minute, hypoxia with oxygen saturation to 82 to 84% on room air, afebrile with normal blood pressure, and negative orthostatic measurements. The patient was initially resuscitated and placed on a non-rebreather with an appropriate response. The review of systems was negative, with no recent travels or sick contacts. Physical exam was notable for decreased breath sounds over the right middle and lower lung bases.

**Figure 1:**
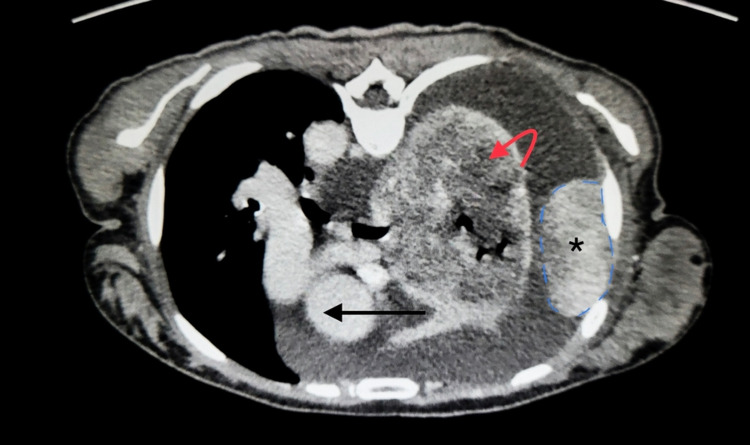
Computed Tomography (CT) of chest with contrast (axial view) showing 6.8x4.1x6.4 cm pleural mass (*) highlighted with blue dashes, with large pleural effusion causing compression atelectasis (red arrow) and mediastinal shift to left (black arrow).

Further investigation showed moderate to large right-sided pleural effusion on chest X-ray. Labs were only significant for mild high sensitivity C-reactive protein elevation and mild leukocytosis with relative lymphocytosis. Multiple differentials were raised, including both infectious and non-infectious etiologies including pneumonia, pulmonary embolism, tuberculosis, and malignancy. Computed tomography (CT) of the chest was performed, which showed a large right-sided enhancing pleural mass along the parietal pleura measuring 6.8 x 4.1 x 6.4 cm along with compression atelectasis from large pleural effusion and mediastinal shift to the left (Figure 1).

Given the clinical context, an abrupt cut-off at the right interlobar bronchus raised suspicion for an endobronchial lesion. Thoracentesis was performed which showed no bacterial, viral or fungal growth on cultures and came back negative for cytology for any malignant cells. 

**Figure 2:**
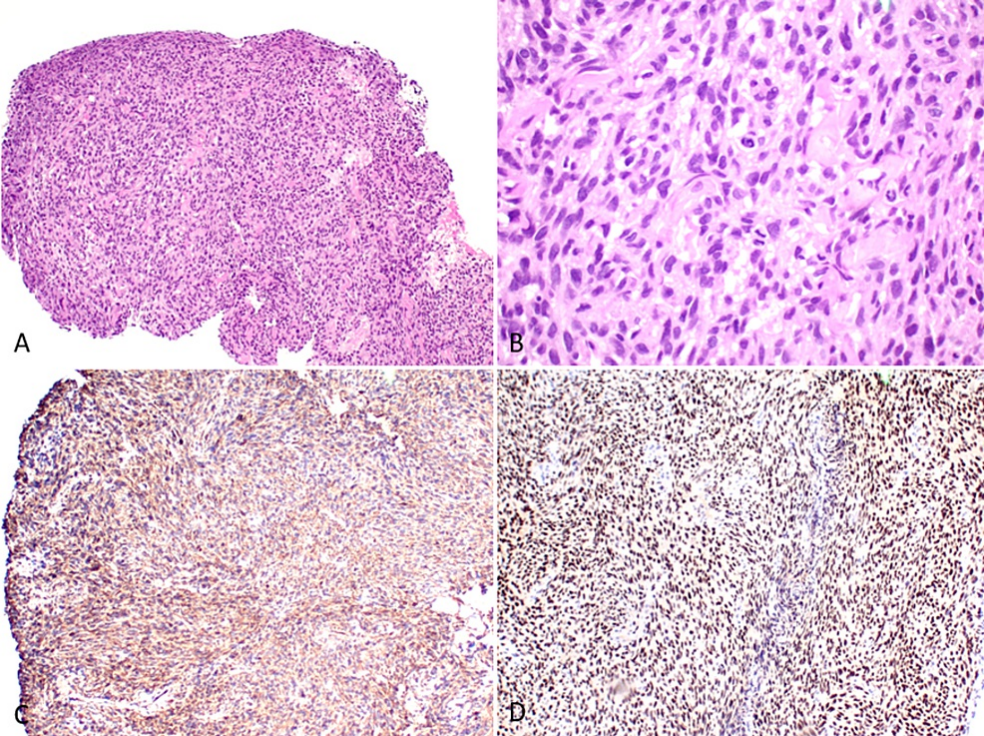
Leiomyosarcoma A. The pleural biopsy shows a neoplasm with sheets of hypercellular spindle cells (H&E, 100×). B. The tumor cells have marked nuclear atypia and brisk mitotic activity (H&E, 400×). They are positive for smooth muscle markers such as C. Caldesmon, and also D. ER immunostaining. H&E: hematoxylin and eosin, ER: estrogen receptor

Thoracoscopy and right pleural biopsy were performed to further understand the underlying etiology. Biopsy revealed high cellular spindle cell proliferation with moderate cytological atypia and brisk mitotic activity showing malignant spindle cell neoplasm. Immunostaining was further done, strongly positive for desmin, h-caldesmon, estrogen and progesterone receptors (ER and PR), Wilms tumor protein (WT-1), with focal positivity for smooth muscle antigen, and calretinin which highlighted the presence of some entrapped mesothelial cells. The morphology and immune profile were most consistent with leiomyosarcoma (LMS) (Figure 2).

A CT scan with contrast for extremities, abdomen, and pelvis was also performed to stage further the tumor, which was negative in pursuit of any concomitant or primary malignancy. Thus, the diagnosis of primary pulmonary leiomyosarcoma (PPL) was made with probable endobronchial origin. For multidisciplinary management, the patient was then referred to thoracic surgery, oncology, and radiation oncology.

## Discussion

We described a case of PPL in a patient who had presented with dyspnea; the clue towards an underlying malignant pathology was apparent from the radiograph. PPL can present with a broad range of unspecific symptoms that range from cough, fever, chest pain, wheezing, hemoptysis, or thoracic symptoms due to tumor expansion into the chest cavity [5]. However, in a retrospective review of 18 patients, most of the patients were asymptomatic [6]. Most of these tumors were discovered during physical and radiographic examination. The radiological findings seen in the radiographs were most often due to bronchial obstruction [7].

Pulmonary sarcomas are most often metastatic from other sites [3]. The challenge in diagnosing a PPL reliably depends on effectively ruling out primary tumors in sites that are more prone to have sarcomas-soft tissues, the uterus, and the gastrointestinal tract [2]. In our study, we obtained CT scans of the extremities, abdomen, and pelvis to look for both primary tumors and metastases. As these imaging studies were unrevealing, we were finally able to confidently diagnose the patient as PPL based on histopathology findings.

PPL may originate within the pulmonary parenchyma, within a bronchus, or within the pulmonary artery [8]. Only the patients with endobronchial involvement were found to be more likely to present with symptoms like hemoptysis, chest pain, or cough [6]. On histopathology, these tumors appear as broad fascicles of malignant cells intersecting perpendicularly which is suggestive of spindle cell proliferation [6]. These tumors also stain positively to desmin and smooth muscle actin-confirming smooth muscle differentiation [8]. Higher-grade tumors stain more focally and weakly, thus making them harder to definitively diagnose.

Surgical resection of the tumor is the preferred modality of treatment [7]. A study of 231 cases of PPL identified distant stage of the tumor and surgical resection as independent prognostic factors; it observed a 57% reduction in the risk of death after complete resection [9]. PPLs rarely metastasize early in the disease; adjuvant chemotherapy or radiotherapy may be used when the tumors are unresectable, are incompletely resected, or are higher grade. Adjuvant therapies, however, were not found to improve survival [10].

## Conclusions

In conclusion, PPLs are extremely rare tumors that may only be apparent for the first time on routine examinations or radiographs. Patients usually present with non-specific symptoms like shortness of breath, cough, hemoptysis, or because of compression of thoracic organs. Definitive diagnosis can be made by histopathology and immunohistochemistry; PPLs stain positively for markers of smooth muscle differentiation. Surgery is the favored modality of treatment. Complete resection of the tumor is associated with a good prognosis.
